# Knowledge and Attitudes towards Antibiotic Use and Resistance - A Latent Class Analysis of a Swedish Population-Based Sample

**DOI:** 10.1371/journal.pone.0152160

**Published:** 2016-04-20

**Authors:** Martina Vallin, Maria Polyzoi, Gaetano Marrone, Senia Rosales-Klintz, Karin Tegmark Wisell, Cecilia Stålsby Lundborg

**Affiliations:** 1 Karolinska Institutet, Department of Public Health Sciences, Tomtebodavägen 18A, Stockholm, Sweden; 2 Public Health Agency of Sweden, Nobels väg 18, Solna, Sweden; Ross University School of Veterinary Medicine, SAINT KITTS AND NEVIS

## Abstract

**Background:**

In 2006, a study investigating knowledge and attitudes regarding antibiotic use and resistance in Sweden, indicated high level of knowledge but also areas in need of improvement.

**Objective:**

(i) To provide an update on the knowledge and attitudes to antibiotic use and resistance of the Swedish population, and (ii) to identify which groups within the population are in particular need of improved knowledge or attitudes.

**Methods:**

A questionnaire was sent by post in 2013 to 2,500 randomly-selected individuals aged 18–74, living in Sweden. Latent class analyses were conducted to group respondents based on their responses. The association between socio-demographic characteristics and the probability of belonging to each latent class was assessed.

**Results:**

The response rate was 57%. Ninety-four per cent of the responders knew that bacteria could become resistant to antibiotics and the majority answered correctly to the questions regarding antibiotic resistance development. The respondents expressed confidence in doctors who decided not to prescribe antibiotics. Three latent classes related to ‘knowledge regarding antibiotic use and resistance’, two regarding ‘attitudes towards antibiotic accessibility and infection prevention’ and three regarding ‘attitudes towards antibiotic use and effects’ were revealed. Men, younger and more educated people were more knowledgeable but males had a less restrictive attitude. Respondents with high levels of knowledge on antibiotics were more likely to have appropriate restrictive attitudes to antibiotics.

**Conclusion:**

Knowledge on antibiotic use and resistance is maintained high and has improved in Sweden compared to 2006. People with lower education and elderly are especially in need of improved knowledge about antibiotic use and resistance.

## Introduction

Antibiotic treatment is a pre-requisite for modern healthcare. Despite the fact that antibiotic resistance is becoming an increasing global health challenge, very few new antibiotics have reached the market in the last 30 years.[1,2] One important measure to minimise the development and spread of resistance is rational use of antibiotics.[1,3] In Europe, antibiotic use varies widely between countries and research shows that overall, countries with high antibiotic consumption have high antibiotic resistance.[4] Although antibiotic use is lower in the Nordic countries than in the rest of Europe,[4] there is still room for improvement. Studies have indicated the occurrence of unnecessary antibiotic prescribing in Sweden[5,6] and there are unexplained differences in antibiotic sales between Swedish counties.[7] In Sweden, national authorities have, together with the network of Strama-groups (The Swedish strategic programme against antibiotic resistance), monitored and analysed national and local resistance and consumption data, and developed recommendations for antibiotic treatment and prophylaxis since 1994. Recurring press events through Swedish media have also been conducted to disseminate information regarding antibiotic resistance development to the general population.[8,9]

In 2006, we conducted a study on knowledge and attitudes regarding antibiotic use and resistance in a random sample of the Swedish population. The results indicated a relatively high level of knowledge. However, some areas needed improvement; in particular, respondents showed uncertainty as to whether antibiotics are indeed effective against viruses.[10]

To further improve antibiotic use and contain resistance through actions such as information campaigns, it is important to have a concrete understanding of the knowledge and attitudes towards antibiotics within different groups of a population, such as age groups, family structure (with or without children), etc. Although antibiotics are prescription-only medicines in Sweden, and this is strictly followed, it is vital that the population is empowered with knowledge on how antibiotics should be used, as well as the risk of resistance. There is a risk that leftover antibiotics may be taken on a later occasion, or given to someone else; alternatively people may consider buying antibiotics abroad or online. Furthermore, understanding attitudes towards antibiotics among the general population is also important; research shows that in cases where patients or patient guardians expect an antibiotic prescription, the likelihood that a doctor will provide a prescription is higher.[11–13] Since our earlier study, no Swedish nationwide population-based studies of knowledge and attitudes towards antibiotic use and resistance have been published.[10] It was therefore decided to repeat the study to: (i) obtain an update on knowledge and attitudes towards antibiotic use and resistance of the population, and (ii) further identify which groups within the population are in particular need of improved knowledge or attitude.

In previous studies analysing knowledge and attitudes, typically either individual items or arbitrary scores have been used. However, we decided to use Latent Class Analysis (LCA) in order to provide additional data on differences in the results from different groups of respondent. LCA is a method which strives to place people into classes based on their responses to items in the questionnaire and not arbitrarily by the researchers.[14]

## Methods

In this population-based cross-sectional study, a questionnaire was sent to 2,500 people between 18 and 74 years of age, selected randomly from the Swedish population using SPAR (statens personadressregister), a governmental register which includes all people residing in Sweden, both Swedish and foreign citizens. SPAR provided a list with selected participants’ names and addresses; data on sex and age were not included.

### Data collection

The questionnaire in English and Swedish (supplementary data), which was developed in Swedish, was based on the questionnaire used in our earlier study.[10] Some questions were revised slightly and tested stepwise for face validity with 17 individuals of different age, sex and professional background. These individuals were not included in the study. The final questionnaire included five areas: (i) antibiotic consumption (8 items), (ii) antibiotic accessibility (6 items), (iii) antibiotic use and its effects (10 items), (iv) side effects and resistance (12 items), and (v) patient experiences, patient-doctor relationships and infection prevention (17 items). The response alternatives were yes/no/don’t know, multiple choice, and a six-point Likert-like scale (where: 1 = totally agree, 6 = do not agree at all and a do not know option denoted 7). A few open-ended questions were also included. Demographic information included questions on sex, age, education and income. The questionnaire did not contain personally identifiable information; instead all participants were given a code to enable reminders. The questionnaires were mailed to the participants’ home address together with an information letter and a pre-paid return envelope. They were sent in February and March 2013 and were collected by the end of April 2013. Two reminders were sent at two weeks intervals, together with new copies of the questionnaire. An information in English for non-Swedish-speaking participants was included, that they could contact the researchers in order to have the questionnaire translated into another language. Sex and age of non-respondents were obtained using authorities’ databases (such as the Swedish tax agency) containing public information about individuals living in Sweden.

### Data management and analysis

Data were entered manually and checked using EpiData 3.1 (EpiData Association, Odense, Denmark). Analyses were carried out using SPSS Software Version 21 (SPSS Inc., Chicago, IL, USA) and the poLCA package for the open-source software R Version 3.0.2 (R Development Core team, 2009).

Descriptive statistics were used to summarise the numerical (mean and standard deviation) and categorical (frequencies and percentages) variables. Cross-tabulation with chi-square tests and independent t-tests were performed to compare sex and mean age respectively, between respondents and non-respondents. The significance level was set to 0.05.

#### Latent class analyses

LCA was used to discover underlying response patterns, thus allowing the identification of respondent groups with similar knowledge and/or attitudes.

Three fields of interest were defined using selected items from the questionnaire for LCA: (i) knowledge regarding antibiotic use and resistance (10 items from the section on side effects and resistance), (ii) attitudes towards antibiotic accessibility and infection prevention (8 items from the sections on antibiotic accessibility and infection prevention), and (iii) attitudes towards antibiotic use and its effects (9 items from the sections on antibiotic use and its effects). The responses to these items (i.e. manifest variables) were used to categorise respondents into groups with similar response profiles (i.e. latent classes). Consequently, the items were selected assuming that more variation in the answers could be observed, rendering them more suitable for a LCA analysis.

Selection of the final LCA models was based on two main criteria: to minimise the values for Akaike Information Criteria (AIC) and Bayesian Information Criteria (BIC), and to obtain a meaningful and interpretable class structure. AIC and BIC are recognised tools for comparing different models in terms of the balance between fit and parsimony.[14] Cases with missing values on the manifest variables were removed (list wise deleted) before estimating the model.

#### Logistic regression analyses

Multinomial and binary logistic regression analyses were conducted to investigate which explanatory variables influenced latent class membership. Odds ratios and 95% confidence intervals were estimated. Groups representing the highest level of knowledge or the most appropriate attitudes were used as reference groups. Respondents who had one or more missing values in the manifest variables were excluded from further analyses.

In addition, the specific item “*I am confident in a doctor’s decision if s/he does not prescribe antibiotics*” (six-point Likert-like scale) was used to create the outcome variable in the ordinal logistic regression model. The new three-level ordinal categorical variable ‘level of confidence’ (with categories labelled as low confidence, medium confidence and high confidence) excluded the ‘do not know’ responses.

Six explanatory variables were used for all the regression analyses. These were: (i) sex, (ii) age (18–29; 30–44; 45–64; 65–74 years), (iii) education (primary and secondary school (or equivalent), upper secondary school (or equivalent), university (or equivalent)), (iv) monthly income (≤14,900; 15,000–25,900; 26,000–40,900; ≥41,000 Swedish crowns (SEK); 1 EURO ≈ 9 SEK), (v) children in the household (none, at least one), and (vi) any medical or healthcare-related education (irrespective of length, which could be as little as a few hours, e.g. a short cardiopulmonary resuscitation course) (yes/no). The ordinal categorical variable ‘knowledge level’ was derived from the LCA model and used as an explanatory variable in the regression models regarding attitudes.

Explanatory variables were tested separately with the significance level set to 0.20 for crude associations, while for the final model the significance level was set to 0.05. The parallel lines test was performed to assess possible violations of the proportional odds assumption. In all the models created, the explanatory variables were tested for collinearity and there was none.

### Ethics

This study was approved by the Regional Ethics Review Board in Stockholm (Registration number: 2012/1993-31).

## Results

### Response rate

The overall response rate was 57% (n = 1426) (Table 1). Women had significantly higher response rates than men (*P*<0.001). The mean age of respondents (48 years) was significantly higher than that of non-respondents (40 years), *P*<0.001. Nine percent (n = 101) of the non-respondents contacted the researchers providing explanations for non-participation. The reasons stated were: did not want to (73), presently abroad (13), unable to answer (10), did not use antibiotics (2), never answered questionnaires (1), perceived the questions as too difficult (1), or having a medical education (1).

**Table 1:** Demographic characteristics of respondents and non-respondents of a population based questionnaire survey in Sweden, 2013.

	Respondents	Non-respondents	
	n (%)	n (%)	
**Total number**	1426 (57%)	1074 (43%)	
**Characteristics**			**p-value**
**Sex**			
Males	622 (43.6)	615 (57.3)	
Females	804 (56.4)	459 (42.7)	<0.001
**Mean Age**	47.8 years	40.2 years	<0.001
**Age groups**			
18–29	226 (16.0)	328 (30.9)	
30–44	363 (25.8)	327 (30.8)	
45–64	563 (40.0)	325 (30.7)	
65–74	257 (18.2)	80 (7.5)	
Missing	17	14	
**Education**			
Primary and secondary school (or equiv.)	220 (15.6)	NA	
Upper secondary school (or equiv.)	539 (38.1)	NA	
University (or equiv.)	655 (46.3)	NA	
Missing	12	NA	
**Income SEK/month** α, β			
≤14 900	354 (26.0)	NA	
15 000–25 900	463 (34.0)	NA	
26 000–40 900	402 (29.6)	NA	
≥41 000	141 (10.4)	NA	
Missing	66	NA	
**At least one child in the household**	462 (32.7)	NA	
**Medical or healthcare-related education**	294 (20.8)	NA	
**Experience of antibiotic use** γ	1061 (94.1)	NA	

Numbers are from this study and SPAR.

NA: Not Accessible.

^α^ The presented options correspond with the options provided in the questionnaire (see supplementary data).

^β^ Exchange rate: 1 EURO ≈ 9 SEK (Swedish crowns).

^γ^ Based on response to the question "Have you ever used antibiotics?".

### Knowledge regarding antibiotic use and resistance

Of the respondents, 94% (n = 1329) knew that bacteria can become resistant to antibiotics (S1 Appendix). A large majority (≥ 90%) answered correctly to the two questions regarding antibiotic resistance development. Fewer respondents (<70%) answered the questions regarding antibiotic side effects correctly, and even fewer (<50%) responded correctly to questions regarding how antibiotic resistance can spread. A minority (12%) answered correctly ‘no’ to the statement, “*People can become resistant to antibiotics”*.

**Fig 1:**
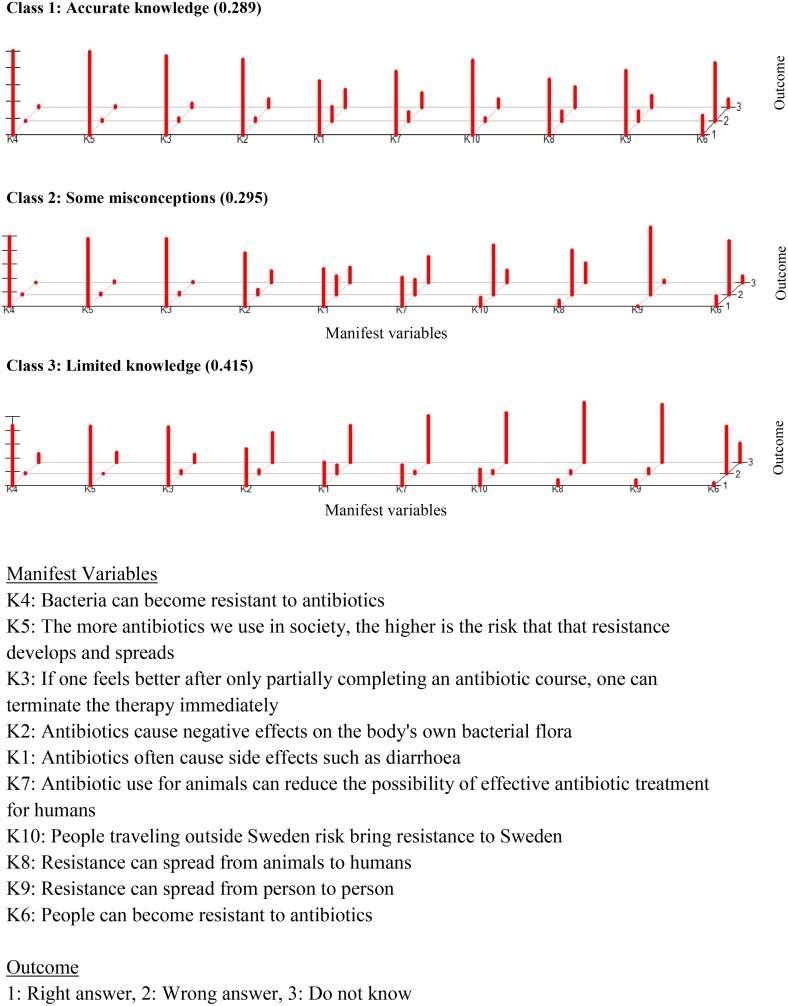
Knowledge regarding antibiotic use and resistance (population based questionnaire survey in Sweden, 2013).

Due to missing values on the manifest variables 41 cases were removed (list wise deleted) before estimating the model, therefore the LCA was based on 1385 responders. A three-class model was chosen to describe patterns on knowledge according to our LCA criteria (Fig 1 and S2 Appendix). The main differences across classes were responses related to antibiotic side effects and how antibiotic resistance spreads. All three classes had similar knowledge related to antibiotic resistance development and adherence to treatment.

Class 1 (n = 390, 29%) was the class with the highest number of correct answers compared to the other classes. This class was labelled ‘*Accurate knowledge’*. Class 2 (n = 404, 29.5%) included respondents who had a higher number of incorrect answers compared to class 1 and 3 and was labelled, ‘*Some misconceptions’*. Older people (>65 years) were more likely to have ‘*Some misconceptions’* than younger people (Table 2). Class 3 (n = 591, 41.5%) included respondents who had a higher number of ‘Do not know’ answers and was therefore labelled, ‘*Limited knowledge’*.

**Table 2:** Descriptive statistics for the variable 'knowledge classes' and multinomial logistic regression analysis of factors influencing knowledge level regarding antibiotics use and resistance among respondents of a population based questionnaire survey in Sweden.

	Knowledge classes			Multinomial logistic regression analysis							
	Accurate knowledge	Some misconceptions	Limited knowledge	Some misconceptions / Accurate knowledge				Limited knowledge / Accurate knowledge			
Characteristics	n (%)	n (%)	n (%)	Crude Ors (95% CI)	sig.	Adjusted ORs (95% CI)	sig.	Crude ORs (95% CI)	sig.	Adjusted ORs (95% CI)	sig.
**Total**	390 (28.2)	404 (29.2)	591 (42.7)								
**Sex**											
Females	198 (25.5)	249 (32.1)	329 (42.4)	1	-	1	-	1	-	1	-
Males	191 (31.7)	151 (25.0)	261 (43.3)	0.6 (0.4–0.8)	0.002	**0.5 (0.3–0.7)**	**<0.001**	0.8 (0.6–1.1)	0.116	**0.6 (0.4–0.9)**	**0.004**
**Age groups**											
18–29	64 (29.2)	46 (21.0)	109 (49.8)	1	-	1	-	1	-	1	-
30–44	109 (30.7)	88 (24.8)	158 (44.5)	1.2 (0.7–2.0)	0.574	1.2 (0.6–2.1)	0.622	0.8 (0.5–1.3)	0.369	1.1 (0.6–1.8)	0.773
45–64	152 (27.8)	178 (32.6)	216 (39.6)	1.7 (1.0–2.8)	0.047	1.7 (1.0–3.1)	0.069	0.8 (0.5–1.2)	0.271	0.9 (0.5–1.5)	0.668
65–74	62 (24.9)	84 (33.7)	103 (41.4)	2.1 (1.2–3.8)	0.012	**2.2 (1.2–4.2)**	**0.017**	0.9 (0.6–1.6)	0.827	0.7 (0.4–1.3)	0.285
**Education**											
Primary and secondary school (or equiv.)	38 (18.2)	52 (24.9)	119 (56.9)	1	-	1	-	1	-	1	-
Upper secondary school (or equiv.)	135 (25.7)	153 (29.1)	238 (45.2)	0.8 (0.5–1.5)	0.626	1.0 (0.6–1.9)	0.951	0.6 (0.3–0.9)	0.023	**0.6 (0.3–1.0)**	**0.043**
University (or equiv.)	214 (33.5)	194 (30.4)	230 (36.1)	0.6 (0.4–1.1)	0.104	0.7 (0.4–1.4)	0.356	0.3 (0.2–0.5)	<0.001	**0.3 (0.2–0.6)**	**<0.001**
**Income (SEK/month)** α											
≤14 900	86 (25.3)	89 (26.2)	165 (48.5)	1	-	1	-	1	-	1	-
15 000–25 900	128 (28.6)	137 (30.6)	183 (40.8)	1.0 (0.7–1.6)	0.909	1.1 (0.7–1.8)	0.745	0.7 (0.5–1.1)	0.134	0.9 (0.6–1.4)	0.537
26 000–40 900	108 (27.5)	129 (32.8)	156 (39.7)	1.2 (0.7–1.8)	0.542	1.4 (0.8–2.3)	0.252	0.7 (0.5–1.1)	0.123	1.0 (0.6–1.6)	0.911
≥41 000	56 (40.3)	34 (24.5)	49 (35.3)	0.6 (0.3–1.1)	0.076	0.8 (0.4–1.6)	0.570	0.4 (0.2–0.7)	0.001	0.7 (0.3–1.2)	0.189
**Children in the household**											
None	257 (27.8)	282 (30.5)	385 (41.7)	1	-	-	-	1	-	-	-
At least one	132 (29.3)	121 (26.9)	197 (43.8)	0.8 (0.6–1.2)	0.267	-	-	1.0 (0.7–1.3)	0.737	-	-
**Medical or healthcare-related education**											
No	269 (24.7)	300 (27.5)	520 (47.8)	1	-	1	-	1	-	1	-
Yes	120 (42.3)	100 (35.2)	64 (22.5)	0.7 (0.5–0.9)	0.021	**0.5 (0.4–0.8)**	**0.001**	0.2 (0.1–0.3)	<0.001	**0.2 (0.1–0.3)**	**<0.001**

^α^ Exchange rate: 1 EURO ≈ 9 (Swedish crowns).

Respondents with higher education were less likely to belong to this group than to ‘*Accurate knowledge’*, compared to those with the lowest educational level. Respondents with any medical or healthcare-related education, as well as men, were less likely to belong to any category other then ‘*Accurate knowledge*’ than women and those without any medical or healthcare-related education. The results do not show any evidence that income, or having at least one child in the household, influences one’s level of knowledge.

### Attitudes towards antibiotic accessibility and infection prevention

Of the responders, 6.1% stated that leftover antibiotics could be saved for personal future use or to give to someone else (data not shown).

**Fig 2:**
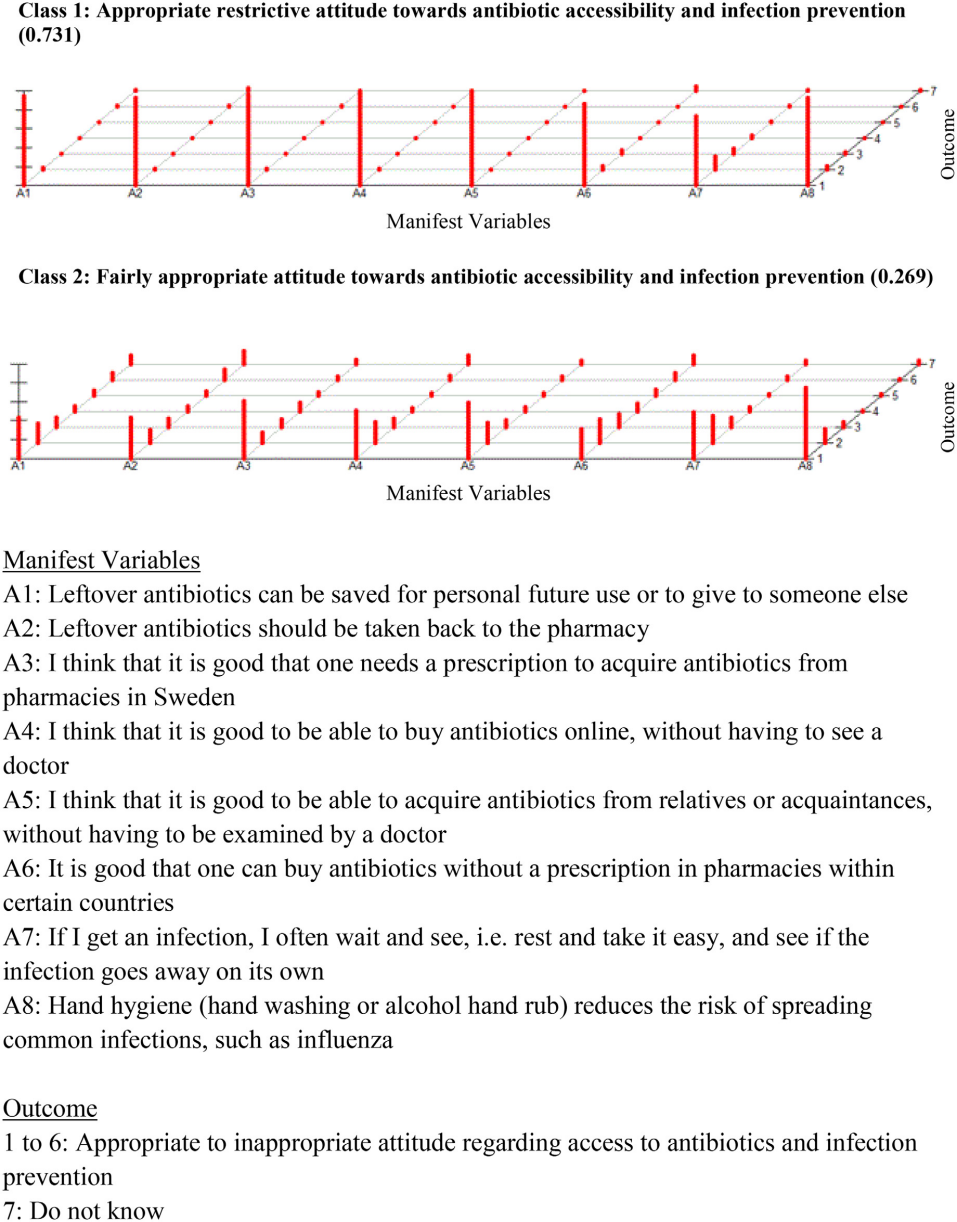
Attitudes towards antibiotic accessibility and infections prevention (population based questionnaire survey in Sweden, 2013).

Due to missing values on the manifest variables 155 cases were removed (list wise deleted) before estimating the model, therefore the LCA was based on 1271 responders. A two-class structure (Fig 2 and S2 Appendix) was chosen when conducting the LCA for attitudes towards antibiotic accessibility (e.g. self-medication) and infection prevention (e.g. hand hygiene). Class 1 (n = 928, 73%), labelled ‘*Appropriate restrictive attitude’*, comprised of respondents who consistently reported a high degree of Appropriate restrictive attitudes regarding how antibiotics should be accessed and how to prevent infections.

Class 2 (n = 343, 27%) was characterised by slightly less consistent appropriate restrictive responses than the first group and was labelled, *‘Fairly appropriate attitude’*. Men and those with ‘*Some misconceptions’* or *‘Limited knowledge’* about antibiotics, were more likely to have a *‘Fairly appropriate attitude’* compared to those with ‘*Accurate knowledge’* (Table 3). People between 45 and 74 years and with any medical or healthcare-related education were less likely to have a ‘*Fairly appropriate attitude’* towards antibiotic accessibility and infection prevention. Similarly, respondents with a monthly income of 15,000–25,900 SEK (significant) and 26,000–40,900 SEK (borderline significant) were less likely to have a *‘Fairly appropriate attitude’* compared to people within the lowest income level.

**Table 3:** Logistic regression analysis of factors associated with appropriate attitude towards access to antibiotics and infection prevention (population based questionnaire survey in Sweden, 2013).

	Logistic regression analysis			
	Fairly appropriate attitude / Appropriate restrictive attitude			
	Appropriate restrictive attitude		Fairly appropriate attitude	
**Total**	928 (73.1%)		343 (26.9%)	
**Characteristics**	**Crude OR(95%CI)**	**sig.**	**Adjusted OR(95%CI)**	**sig.**
**Sex**				
Females	1	-	1	-
Males	1.3 (0.9–1.7)	0.121	1.5 (1.1–2.1)	**0.022**
**Age groups**				
18–29	1	-	1	-
30–44	0.5 (0.3–0.7)	<0.001	0.7 (0.4–1.2)	0.169
45–64	0.2 (0.1–0.3)	<0.001	0.3 (0.2–0.4)	**<0.001**
65–74	0.1 (0.0–0.1)	<0.001	0.1 (0.0–0.1)	**<0.001**
**Education**				
Primary and secondary school (or equiv.)	1	-	1	-
Upper secondary school (or equiv.)	1.4 (0.9–2.2)	0.182	0.7 (0.4–1.3)	0.289
University (or equiv.)	1.4 (0.9–2.2)	0.197	0.8 (0.5–1.5)	0.519
**Income (SEK/month)**α				
≤14 900	1	-	1	-
15 000–25 900	0.6 (0.4–0.9)	0.024	0.6 (0.4–1.0)	**0.036**
26 000–40 900	0.6 (0.4–0.9)	0.021	0.6 (0.4–1.0)	0.060
≥41 000	0.8 (0.5–1.3)	0.347	0.7 (0.4–1.4)	0.360
**Children in the household**				
None	1	-	1	-
At least one	1.3 (0.9–1.7)	0.115	0.8 (0.5–1.1)	0.141
**Medical or healthcare-related education**				
No	1	-	1	-
Yes	0.5 (0.3–0.7)	<0.001	0.6 (0.4–0.9)	**0.026**
**Knowledge class**				
Accurate knowledge	1	-	1	-
Some misconceptions	1.2 (0.8–1.9)	0.334	1.7 (1.1–2.7)	**0.018**
Limited knowledge	2.0 (1.4–2.8)	<0.001	1.9 (1.2–2.9)	**0.003**

^α^ Exchange rate: 1 EURO ≈ 9 SEK (Swedish crowns).

### Attitudes towards antibiotic use and its effects

Of the respondents, 13.4% and 29.5% stated incorrectly that antibiotics make people recover faster when having a cold and that ear infections in a 3–6 year old child always need to be treated with antibiotics, respectively (data not shown).

**Fig 3:**
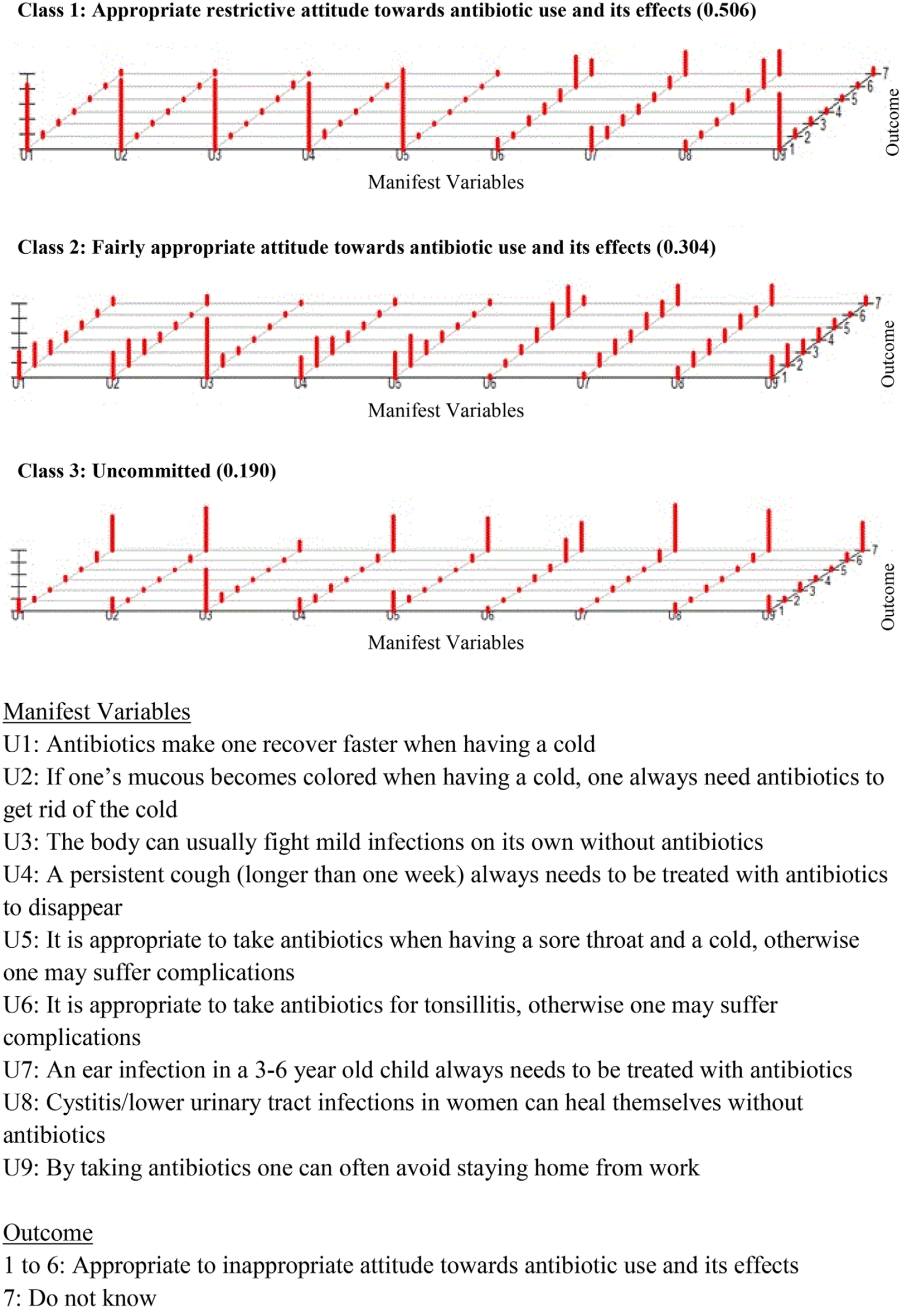
Attitudes towards antibiotic use and its effects (population based questionnaire survey in Sweden, 2013.)

Due to missing values on the manifest variables 164 cases were removed (list wise deleted) before estimating the model, therefore the LCA was based on 1262 responders. The most appropriate LCA model describing attitudes towards antibiotic use and antibiotics effects consisted of three classes (Fig 3 and S2 Appendix). Classes 1 (n = 638, 51%) and 2 (n = 384, 30%) were labelled, ‘*Appropriate restrictive attitude’* and *‘Fairly appropriate attitude’*, respectively. These groups had almost equivalent appropriate responses in relation to antibiotic use for symptoms indicating common cold or flu. Furthermore, both classes had responses which were not consistent with evidence-based recommendations for antibiotic use for specific infections, e.g. tonsillitis, ear infections in children and urinary tract infections in women.[15] However, respondents with an *‘Appropriate restrictive attitude’* reported a more restrictive approach in antibiotic use for sore throat and cough. Class 3 (n = 240, 19%) consisted of respondents with the highest number of ‘do not know’ answers. This class was labelled ‘*Uncommitted’*.

Male respondents were more likely to be *‘Uncommitted’* than to demonstrate an ‘*Appropriate restrictive attitude’* towards antibiotic use and its effects than female respondents (Table 4). People with university-level education (significant), at least 15,000 SEK/month (borderline significant), at least one child in the household, or any medical or healthcare-related education, were less likely to be ‘*Uncommitted*’ than other respondents. In contrast, respondents who had ‘*Some misconceptions’* or *‘Limited knowledge’* on antibiotics, were more likely to be ‘*Uncommitted’* and to have a *‘Fairly appropriate attitude’* towards antibiotic use and its effects, than those with ‘*Accurate knowledge’*.

**Table 4:** Multinomial logistic regression analysis of factors associated with attitude towards the use and effects of antibiotics (population based questionnaire survey in Sweden, 2013)

	Multinomial logistic regression analysis							
	Fairly appropriate attitude / Appropriate restrictive attitude			Uncommitted / Appropriate restrictive attitude				
	Appropriate restrictive attitude			Fairly appropriate attitude		Uncommitted		
**Total**	638 (50.6%)			384 (30.4%)		240 (19.0%)		
**Characteristics**	**Crude ORs (95%CI)**	**sig.**	**Adjusted ORs(95%CI)**	**sig.**	**Crude ORs (95% CI)**	**sig.**	**AdjustedORs(95%CI)**	**sig.**
**Sex**								
Females	1	-	1	-	1	-	1	-
Males	0.9 (0.7–1.2)	0.403	1.0 (0.7–1.4)	0.985	1.5 (1.0–2.1)	0.041	1.9 (1.2–2.9)	**0.006**
**Age groups**								
18–29	1	-						
30–44	0.7 (0.5–1.2)	0.181	1.2 (0.7–2.0)	0.629	0.5 (0.3–0.8)	0.005	1.6 (0.8–3.1)	0.196
45–64	0.9 (0.6–1.4)	0.739	1.3 (0.8–2.2)	0.338	0.6 (0.4–1.0)	0.054	1.2 (0.6–2.4)	0.548
65–74	0.9 (0.6–1.6)	0.828	0.9 (0.5–1.7)	0.775	1.5 (0.9–2.5)	0.169	1.4 (0.7–2.9)	0.327
**Education**								
Primary and secondary school (or eqviv.)	1	-	1	-	1	-	1	-
Upper secondary school (or eqviv.)	0.8 (0.5–1.2)	0.219	0.7 (0.4–1.3)	0.261	0.4 (0.2–0.6)	<0.001	0.7 (0.4–1.2)	0.184
University (or eqviv.)	0.5 (0.3–0.8)	0.009	0.6 (0.4–1.1)	0.089	0.2 (0.1–0.3)	<0.001	0.4 (0.2–0.7)	**0.002**
**Income (SEK/month)** α								
≤14 900	1	-	1	-	1	-	1	-
15 000–25 900	0.7 (0.5–1.1)	0.126	0.7 (0.5–1.1)	0.107	0.5 (0.3–0.8)	0.004	0.6 (0.3–1.0)	0.060
26 000–40 900	0.6 (0.4–0.9)	0.024	0.7 (0.4–1.1)	0.088	0.4 (0.2–0.6)	<0.001	0.4 (0.2–0.8)	**0.009**
≥41 000	0.5 (0.3–0.9)	0.024	0.7 (0.4–1.2)	0.188	0.2 (0.1–0.5)	<0.001	0.5 (0.2–1.2)	0.095
**Children in the household**								
None	1	-	1	-	1	-	1	-
At least one	0.8 (0.6–1.1)	0.120	0.8 (0.6–1.2)	0.363	0.4 (0.3–0.6)	<0.001	0.4 (0.2–0.7)	**0.001**
**Medical or healthcare-related education**								
No	1	-	1	-	1	-	1	-
Yes	0.8 (0.5–1.1)	0.105	0.9 (0.6–1.3)	0.463	0.2 (0.1–0.4)	<0.001	0.3 (0.1–0.6)	**0.001**
**Knowledge class**								
Accurate knowledge	1	-	1	-	1	-	1	-
Some misconceptions	1.8 (1.3–2.6)	0.001	1.8 (1.2–2.6)	**0.004**	2.2 (1.2–4.3)	0.017	2.3 (1.1–4.7)	**0.022**
Limited knowledge	2.1 (1.5–3.1)	<0.001	2.0 (1.4–3.0)	**<0.001**	10.5 (5.9–18.6)	<0.001	8.5 (4.6–15.8)	**<0.001**

^α^ Exchange rate: 1 EURO ≈ 9 SEK (Swedish crowns).

### Confidence in a doctor’s decision not to prescribe antibiotics

Of the responders, 79.2% expressed confidence (three first levels of agreement in 6-level scale of agreement) in doctor’s decision to not prescribe antibiotics, while 88.7% expressed confidence in doctor’s decision to prescribe antibiotics (data not shown).

After initial cross-tabulation, the explanatory variable ‘monthly income’ was grouped into three categories (≤14,900 SEK, 15,000–25,900 SEK and ≥26,000 SEK) due to the low frequencies observed in the four original categories. The analysis showed that younger respondents (significant) and women (borderline significant) were less likely to have high confidence in a doctor’s decision not to prescribe antibiotics than older respondents (S3 Appendix). The parallel lines test showed that there was no violation of the proportional odds assumption (*P* = 0.389).

## Discussion

Our results show that the Swedish population’s level of knowledge about antibiotic use and resistance represented by the participants has not only remained high but has also increased since 2006. A decrease was observed in the percentage of people who agreed to the wrong statement that antibiotics make one recover faster when having a cold (19.1% in 2006 and 13.4% in 2013), that ear infections in a 3–6 year old child always need to be treated with antibiotics (49.5% in 2006 and 29.5% in 2013) and that people become resistant to antibiotics (84.7% in 2006 and 70.8% in 2013). Regarding whether bacteria can become resistant to antibiotics the percentage of respondents who agreed increased from 80.7% in 2006 to 93.7% in 2013.

Similarly, a recent European survey showed that Swedish respondents had the highest knowledge compared to other European participants about antibiotic ineffectiveness against viruses (77% vs EU27 average of 40%) and common colds (77% vs EU27 average of 52%).[16] Previous studies from the United Kingdom, Germany and the Netherlands have reported lower knowledge levels among the general public in relation to similar questions, as compared to our results.[17–19] The results of our study show that the majority of respondents had an appropriate and restrictive attitude towards antibiotics. The improvement in knowledge about antibiotics since 2006 could be due to campaigns in the connection with the “European Antibiotic Awareness Day” that started in 2008. This day is organized by European Centre for Disease Prevention and Control every year and aims to spread up-to-date knowledge regarding appropriate antibiotic use to the public and to health care staff.[20] Strama’s and other agencies’ extensive work in Sweden since 2006 could also have had a positive effect on the level of knowledge among the general population.[21] For example, in connection with European Antibiotic Awareness Day, Strama developed a brochure with information regarding when you need antibiotics, why antibiotics do not help against common colds etc.[22] This brochure can be found at many health centers in Sweden.

Although respondents in the present study understood the relationship between unnecessary antibiotic use and antibiotic resistance development, some misconceptions still exist. In the 2006 survey,[10] confusion concerning the term ‘antibiotic resistance’ was detected; 85% agreed with the statement “*People can become resistant to antibiotics*”. In our current study, this has improved, however it still requires attention. This misinterpretation of antibiotic resistance has been confirmed and explored further in qualitative studies elsewhere,[23,24] highlighting the importance of providing clear messages from healthcare workers and awareness campaigns. A better understanding of the biomedical concept of antibiotic resistance might encourage individuals to take responsibility, and the general public to engage in practising behaviours aimed at containing spread of antibiotic resistance, for the benefit of society.

Overall, respondents had a comprehensive knowledge-base, in line with the main messages promoted by Strama[9] and other governmental initiatives.[25] The knowledge gaps identified in this study relate mainly to how antibiotic resistance spreads. This finding underlines the importance of incorporating information about routes of spread of resistant bacteria between individuals as well as between different sectors, as emphasised in the One Health concept;[26] taking a more comprehensive approach when developing new messages.

We were able to characterise the underlying respondents’ profiles by using LCA. To our knowledge, this is the first time that this approach has been used to analyse knowledge and attitudes towards antibiotic use and resistance. This characterisation will facilitate the segmentation and subsequent development of tailored messages in future campaigns. For instance, in our study, male respondents were more knowledgeable about antibiotics than women, whereas the Swedish study from 2006,[10] a British, a Spanish and a Dutch study showed the opposite.[18,19,27] None of the afore mentioned studies utilised LCA. Nevertheless, whilst men had more knowledge about antibiotics than women, they were more likely to have only a ‘*Fairly appropriate’* attitude towards antibiotic accessibility and infection prevention (for explanation see methods section). Other research supports this finding by showing that males were more likely to report that they behaved against recommendations regarding antibiotic use.[28–30] We also found that the younger participants had higher knowledge than the older ones. This result was similar to a Korean study which showed that younger participants had more knowledge.[31] Conversely, a Malaysian study showed that younger participants were less knowledgeable than older counterparts.[32] Furthermore, older people were less likely than younger people to have a ‘*Fairly appropriate attitude’* towards antibiotic accessibility and infection prevention. Similar results were seen in a Malaysian study.[28] Respondents with ‘*Accurate knowledge’* about antibiotics were more likely to have an ‘*Appropriate restrictive’* attitude towards antibiotics. A comparable relationship between knowledge and attitudes was also seen in the studies from Malaysia and South Korea.[28,31]

In addition to knowledge level, the cultural constructs prevalent in a society also play a role in the patterns of antibiotic use. Previous studies have shown that European countries where particular cultural characteristics, as defined by Hoftede’s model, are more prone to excessive use of antibiotics.[33,34] These cultural dimensions include a strong sense of hierarchy (power distance), low tolerance to ambiguity (uncertainty avoidance) and predominance of assertiveness and competitiveness in decision-making processes (masculinity). In contrast, Sweden exhibits a different cultural profile that favours a more prudent approach towards antibiotic use in general. Swedish society is characterised by preference for deliberation and consensus (i.e. low power distance); high adaptability to unknown situations (low uncertainty avoidance) and has a rather low masculinity score. Interesting enough, the high level of individualism observed among Swedes could be related to a preference for self-medication, following this cultural approach. However, a fairly low number of respondents (6%) expressed their interest in using left over antibiotics. Even so, attention must be given to new channels of accessing antibiotics, such travel and online purchases.

A large majority of the respondents had high confidence in a doctor’s decision not to prescribe antibiotics. Comparable results were observed in Germany, where in a web-based survey, the majority of respondents said that they would trust their doctor if s/he chose not to prescribe antibiotics.[17] Divergent results were reported recently in a practice-level study from England, where patient satisfaction ratings decreased when antibiotic prescribing was lower than the national mean.[35] However, the aforementioned report did not directly evaluated the relationship between satisfaction and antibiotic prescribing at individual level as it was assessed in the current report. In our study, trust in doctors when antibiotics are not prescribed decreased since 2006.[10] Interestingly, trust in doctors who prescribe antibiotics increased.[10] This phenomenon might be the result of a well-informed, highly-aware society, with access to alternative sources of information (e.g. internet) combined with rather restricted access to antibiotics within the Swedish healthcare system. The Swedish Medical Products Agency has reported an increase in the number of internet searches on how to buy medicines online, particularly antibiotics for *Chlamydia* infections.[36] Younger people were identified as a risk group for this behaviour, which is consistent with our findings that younger respondents were less likely to trust a doctor’s decision not to prescribe antibiotics. This finding stresses the importance of establishing good communication between patients and healthcare providers, particularly within this population segment.

### Methodological considerations

This study has several strengths, including a large sample size randomly selected from a population-based sampling frame, with a relatively high response rate. The results could potentially be transferable also to other similar populations. In addition, we used a previously tested questionnaire, which contributed to the validity of our study and allowed for comparison with previous results from the same population. The use of LCA allowed us to cluster participants according to the similarity of their answers, instead of using arbitrary scores. Furthermore, LCA enabled the identification of population groups, which can be targeted through future tailored interventions.

One of the study’s limitations was potential overestimation of positive outcomes. It is possible, that people who were more informed or interested in the topic were more willing to participate. Males and young people were under-represented among the respondents, potentially influencing the results. The lower number of men responding is not due to changes in the population, since the number of men has increased in Sweden since 2006.[37] There could also be a negative selection bias for non-Swedish speaking people, meaning that a lower proportion of non-Swedish speaking people responded to the questionnaire. Potentially, the percentage of respondents with a university degree, could have led to an overestimation of the good knowledge observed in this study. There could also be a selection bias considering that 20% has stated that they have a medical or healthcare-related education. This should be taken in to consideration when interpreting the relationship between high education, accurate knowledge and appropriate restrictive attitude to antibiotics. However, a large proportion of those who stated that they had medical or healthcare-related education had only taken a very short cardiopulmonary resuscitation course or the like.

In addition to that, another limitation of the current study is that the LCA models have not been modified to take into account for potential violation of the conditional dependence assumption and the p-values were not adjusted to avoid the issue of multiplicity. However, other authors have reported that even the conditional dependence can be technically incorrect and the results of a LCA with conditional dependence can provide estimates close to the values of the technically correct conducted LCA but with more meaningful and easier interpretable results.[38, 39]

Due to logistical reasons, it was not possible to conduct telephone interviews as in 2006.[10] Although the mailed questionnaires might have minimised the social desirability effect, there is no certainty that the participants did not search for the answers, asked someone else, or that the questionnaire was actually answered by the selected person. Another shortcoming when using a postal questionnaire is that participants do not have the possibility to ask the interviewer for clarification in cases where questions may have been unclear to them.

## Conclusion and Implications

The Swedish population’s level of knowledge and awareness regarding antibiotic use and resistance is high compared to other European countries and has increased since 2006. The results indicate that a high level of knowledge regarding antibiotics has a positive effect on attitudes towards antibiotics. People with lower levels of education and elderly people are especially in need of improved knowledge about antibiotics.

Future actions should aim to maintain the already high level of knowledge among the Swedish population and focus on improving knowledge regarding the spread of antibiotic resistance. These actions should target groups who have more misconceptions about antibiotic use and resistance, such as older people and people with lower educational levels, although no group should be excluded when planning information initiatives. Finally, the “Swedish success story” in building knowledge and sustaining appropriate attitudes among public and the parameters that led to it should be further investigated in order to identify the potential of using similar approaches in other settings where the problem of antibiotic use and resistance is higher.

## Supporting Information

S1 Appendix Respondents’ knowledge about antibiotic use and resistance. (DOC)

S2 Appendix Criteria for choosing the number of classes in the latent class analysis. (DOC)

S3 Appendix Ordinal logistic regression analysis of factors associated with respondents' confidence in doctors' decision to not prescribe antibiotics. (DOC)

S4 Appendix Questionnaire on antibiotics. (PDF)

S5 Appendix Enkät om antibiotika. (PDF)

S1 File Attitude 1. (R)

S2 File Attitude 2. (R)

S3 File Data set. (XLS)

S4 File Knowledge regression. (R)

S5 File Latent class analysis. (R)
