# Supplementary material for: Knowledge and Attitudes towards Antibiotic Use and Resistance - A Latent Class Analysis of a Swedish Population-Based Sample
Source: PLoS One. 2016 Apr 20;11(4):e0152160. doi: 10.1371/journal.pone.0152160 (PMC4838333; doi:10.1371/journal.pone.0152160)
Supplement: S1 Appendix — (DOC) [file pone.0152160.s001.doc]

# S1 Appendix. Respondents’ knowledge about antibiotic use and resistance

Respondents’ knowledge about antibiotic use and resistance by age group and sex (population based questionnaire survey in Sweden, 2013)

|  | **Males** | | | | | **Females** | | | | | **All respondents** |
| --- | --- | --- | --- | --- | --- | --- | --- | --- | --- | --- | --- |
|  | 18-29 | 30-44 | 45-64 | 65-74 | Total | 18-29 | 30-44 | 45-64 | 65-74 | Total | Total |
|  | n=98 | n=156 | n=246 | n=116 | n=616 | n=128 | n=207 | n=316 | n=141 | n=792 | n=1408 |
| **Statements** (correct answer) | % | % | % | % | % | % | % | % | % | % | % |
| 1. Bacteria can become resistant to antibiotics (Yes) | 89 | 94 | 95 | 94 | 94 | 87 | 92 | 96 | 96 | 94 | 94 |
| 2. The more antibiotics we use in society, the higher is the risk that resistance develops and spreads (Yes) | 80 | 96 | 93 | 93 | 92 | 87 | 91 | 94 | 90 | 91 | 92 |
| 3. If one’s feels better after only partially completing an antibiotic course, one can terminate the therapy immediately (No) | 71 | 86 | 92 | 90 | 87 | 89 | 93 | 96 | 93 | 93 | 90 |
| 4. Antibiotics cause negative effects on the body's own bacterial flora (Yes) | 59 | 66 | 68 | 70 | 67 | 62 | 70 | 76 | 71 | 71 | 69 |
| 5. Antibiotics often cause side effects such as diarrhoea (Yes) | 34 | 35 | 43 | 49 | 41 | 41 | 45 | 60 | 60 | 53 | 48 |
| 6. Antibiotic use for animals can reduce the possibility of effective antibiotic treatment for humans (Yes) | 39 | 56 | 51 | 47 | 50 | 34 | 44 | 43 | 41 | 41 | 45 |
| 7. People traveling outside Sweden risk bringing resistance to Sweden (Yes) | 43 | 43 | 39 | 50 | 43 | 36 | 37 | 37 | 35 | 37 | 39 |
| 8. Resistance can spread from animals to humans (Yes) | 29 | 33 | 28 | 29 | 30 | 19 | 24 | 21 | 17 | 21 | 25 |
| 9. Resistance can spread from person to person (Yes) | 31 | 31 | 27 | 21 | 28 | 27 | 26 | 22 | 18 | 23 | 25 |
| 10. People can become resistant to antibiotics (No) | 12 | 20 | 15 | 7 | 14 | 12 | 14 | 10 | 7 | 11 | 12 |
