# Supplementary material for: Knowledge and Attitudes towards Antibiotic Use and Resistance - A Latent Class Analysis of a Swedish Population-Based Sample
Source: PLoS One. 2016 Apr 20;11(4):e0152160. doi: 10.1371/journal.pone.0152160 (PMC4838333; doi:10.1371/journal.pone.0152160)
Supplement: S2 Appendix — (DOC) [file pone.0152160.s002.doc]

# S2 Appendix. Criteria for choosing the number of classes in the latent class analysis

For the knowledge assessment ten questions regarding antibiotic use and resistance were used (figure 1). The AIC and BIC were minimised using four levels of knowledge (AIC: 18501.91 and BIC: 18936.29). However, with three levels of knowledge the differences in AIC and BIC were small (AIC: 18969.02 and BIC: 19293.5). Thus, for practical reasons three levels of knowledge were preferred.

In order to assess the respondents’ attitudes towards access to antibiotics and infection prevention, eight relevant questions were selected from the questionnaire (figure 2). As the number of classes was increasing from two to six, the AIC was slightly decreasing [AIC(2): 16059.82, AIC(6): 15668.75], while the BIC was slightly increasing [BIC(2): 16567.53, BIC(6): 17202.36]. Thus, for practical reasons two classes were preferred.

To assess the respondents’ attitudes towards the use and effects of antibiotics, nine relevant questions were selected from the questionnaire (figure 3). As the number of classes was increasing from two to six, the AIC was slightly decreasing [AIC(2): 33673.81, AIC(6): 31903.17]. Similarly, the BIC was slightly decreasing when the number of classes was increasing from two to four, but it was increasing with five and six classes. Thus, even though the two criteria were minimized using four classes [AIC(4): 32164.48, BIC(4): 33309.5], the three classes option was preferred, since the difference was too small [AIC(3): 32552.89, BIC(3): 33410.35] and it reduced the complexity of the classes description.

In order to define which beliefs constitute an appropriate attitude, the recommendations for drug use for common diseases were used.^15^The answers to the questions regarding use and effects of antibiotics were checked for their consistency with these recommendations.
