# Supplementary material for: Knowledge and Attitudes towards Antibiotic Use and Resistance - A Latent Class Analysis of a Swedish Population-Based Sample
Source: PLoS One. 2016 Apr 20;11(4):e0152160. doi: 10.1371/journal.pone.0152160 (PMC4838333; doi:10.1371/journal.pone.0152160)
Supplement: S3 Appendix — (DOC) [file pone.0152160.s003.doc]

# S3 Appendix. Ordinal logistic regression analysis of factors associated with respondents' confidence in doctors' decision to not prescribe antibiotics

Ordinal logistic regression analysis of factors associated with respondents' confidence in doctors' decision to not prescribe antibiotics (population based questionnaire survey in Sweden, 2013)

| **Characteristics** | **Crude ORs (95% CI)** | **p-value** | **Adjusted ORs (95% CI)** | **p-value** |
| --- | --- | --- | --- | --- |
| **Sex** |  |  |  |  |
| Women | 0.8 (0.6-1.0) | 0.032 | 0.8 (0.6-1.0) | **0.059** |
| Men | 1 | - | 1 | - |
| **Age groups** |  |  |  |  |
| 18 – 29 | 0.3 (0.2-0.5) | <0.001 | 0.3 (0.2-0.5) | **<0.001** |
| 30 – 44 | 0.3 (0.2-0.5) | <0.001 | 0.3 (0.2-0.5) | **<0.001** |
| 45 – 64 | 0.4 (0.3-0.7) | <0.001 | 0.4 (0.3-0.7) | **<0.001** |
| 65 – 74 | 1 | - | 1 | - |
| **Education** |  |  |  |  |
| Primary and secondary school (or equiv.) | 1.2 (0.9-1.8) | 0.273 | - | - |
| Upper secondary school (or equiv.) | 1.0 (0.8-1.3) | 0.965 | - | - |
| University (or equiv.) | 1 | - | - | - |
| **Income (SEK/month)**α |  |  |  |  |
| ≤14 900 | 1.0 (0.7-1.3) | 0.819 | - | - |
| 15 000 - 25 000 | 0.9 (0.7-1.2) | 0.451 | - | - |
| ≥26 000 | 1 | - | - | - |
| **Children in the household** |  |  |  |  |
| None | 1.4 (1.1-1.8) | 0.004 | 1.1 (0.8-1.4) | 0.635 |
| At least one | 1 | - | 1 | - |
| **Medical or healthcare-related education** |  |  |  |  |
| No | 1.0 (0.7-1.3) | 0.897 |  |  |
| Yes | 1 | - | - | - |
| **Knowledge regarding antibiotics effects and resistance** |  |  |  |  |
| Accurate knowledge | 1.2 (0.9-1.6) | 0.237 | - | - |
| Some misconceptions | 1.1 (0.9-1.5) | 0.373 | - | - |
| Limited knowledge | 1 | **-** | **-** | **-** |

α Exchange rate: 1 EURO ≈ 9 SEK (Swedish crowns)
