# Supplementary material for: Knowledge and Attitudes towards Antibiotic Use and Resistance - A Latent Class Analysis of a Swedish Population-Based Sample
Source: PLoS One. 2016 Apr 20;11(4):e0152160. doi: 10.1371/journal.pone.0152160 (PMC4838333; doi:10.1371/journal.pone.0152160)
Supplement: S4 Appendix — (PDF) [file pone.0152160.s004.pdf]

Questionnaire on antibiotics

1. Antibiotic consumption

Antibiotics are medications which are sometimes used to treat infections. There are several different types of antibiotics; penicillin is the most common.

1.1 Please name some of the antibiotics you have heard of below.

____

____

1.2 Which of the following medications are antibiotics? *Please tick one or more options.*

☐ Paracetamol	☐ Bricanyl
☐ Seloken	☐ Selexid
☐ Kåvepenin	☐ Alvedon

1.3 Have you ever taken antibiotics? *Please tick only one option.*

☐ Yes
☐ No ➔ *Proceed to 1.5*
☐ Don't know ➔ *Proceed to 1.5*

1.4 How many times have you consumed antibiotics during the past 12 months? *Please tick only one option.*

☐ Never
☐ Once
☐ 2-5 times
☐ More than 5 times

1.5 How many times has another adult in your household (over 18 years old) received antibiotics during the past 12 months? *Please tick only one option.*

☐ Never ☐ Don't know
☐ Once
☐ 2-5 times
☐ More than 5 times
☐ There are no other adults in the household

1.6 How many children under the age of 18 live in your household on a regular basis (part-time or more)? *Please tick only one option.*

☐ 0 ➔ *Proceed to 1.8*
☐ 1
☐ 2
☐ 3 or more

1.7 How many times have children in your household received antibiotics during the past 12 months (in total)? *Please tick only one option.*

☐ Never ☐ Don't know
☐ Once
☐ 2-5 times
☐ More than 5 times

1.8 Is anyone in your household taking antibiotics at the moment? *Please tick only one option.*

☐ Yes, for what reason? ___________________________ Please specify the person's age _____ years
☐ No
☐ Don't know

2. Antibiotic accessibility

The following questions should be answered using the scale below. Use this scale to indicate to what extent you agree with the question/statement. Please tick only one option.

	Totally agree					Don't agree at all	Don't know
2.1 Leftover antibiotics can be saved for personal future use or to give to someone else.	☐	☐	☐	☐	☐	☐	☐
2.2 Leftover antibiotics should be taken back to the pharmacy.	☐	☐	☐	☐	☐	☐	☐
2.3 I think that it is good that one needs a prescription to acquire antibiotics from pharmacies in Sweden.	☐	☐	☐	☐	☐	☐	☐
2.4 I think that it is good to be able to buy antibiotics online, without having to see a doctor.	☐	☐	☐	☐	☐	☐	☐
2.5 I think that it is good to be able to acquire antibiotics from relatives or acquaintances, without having to be examined by a doctor.	☐	☐	☐	☐	☐	☐	☐
2.6 It is good that one can buy antibiotics without a prescription in pharmacies within certain countries.	☐	☐	☐	☐	☐	☐	☐

3. Antibiotic use and its effects

	Totatally agree					Don't agree at all	Don't know
3.1 Antibiotics make one recover faster when having a cold.	☐	☐	☐	☐	☐	☐	☐
3.2 If one's mucous becomes coloured when having a cold, one always need antibiotics to get rid of the cold.	☐	☐	☐	☐	☐	☐	☐
3.3 The body can usually fight mild infections on its own without antibiotics.	☐	☐	☐	☐	☐	☐	☐
3.4 A persistent cough (longer than one week) always needs to be treated with antibiotics to disappear.	☐	☐	☐	☐	☐	☐	☐
3.5 It is appropriate to take antibiotics when having a sore throat and a cold, otherwise one may suffer complications.	☐	☐	☐	☐	☐	☐	☐
3.6 It is appropriate to take antibiotics for tonsillitis, otherwise one may suffer complications.	☐	☐	☐	☐	☐	☐	☐
3.7 An ear infection in a 3-6 year old child always needs to be treated with antibiotics.	☐	☐	☐	☐	☐	☐	☐
3.8 Cystitis/lower urinary tract infections in women can heal themselves without antibiotics.	☐	☐	☐	☐	☐	☐	☐
3.9 By taking antibiotics one can often avoid staying home from work.	☐	☐	☐	☐	☐	☐	☐
3.10 Antibiotics are supposed to kill all bacteria in the body.	☐	☐	☐	☐	☐	☐	☐

4. Side effects and resistance

	Yes	No	Don't know
4.1 Antibiotics often cause side effects such as diarrhoea.	☐	☐	☐
4.2 Antibiotics cause negative effects on the body's own bacterial flora.	☐	☐	☐
4.3 If one's feels better after only partially completing an antibiotic course, one can terminate the therapy immediately.	☐	☐	☐
4.4 Bacteria can become resistant to antibiotics.	☐	☐	☐
4.5 The more antibiotics we use in society, the higher is the risk that resistance develops and spreads.	☐	☐	☐
4.6 People can become resistant to antibiotics.	☐	☐	☐

	Yes	No	Don't know
4.7 Antibiotic use for animals can reduce the possibility of effective antibiotic treatment for humans.	☐	☐	☐
4.8 Resistance can spread from animals to humans.	☐	☐	☐
4.9 Resistance can spread from person to person.	☐	☐	☐
4.10 People travelling outside Sweden risk bringing resistance to Sweden.	☐	☐	☐

	Totally agree					Don't agree at all	Don't know
4.11 Today, antibiotic resistance is a big problem in Sweden.	☐	☐	☐	☐	☐	☐	☐
4.12 Today, antibiotic resistance is a big problem in the world.	☐	☐	☐	☐	☐	☐	☐

5. Patient experiences, patient-doctor relationships and infection prevention

	Totally agree					Don't agree at all	Don't know
5.1 Doctors always conduct a thorough examination regarding whether a patient is in need of antibiotics or not.	☐	☐	☐	☐	☐	☐	☐
5.2 Doctors prescribe antibiotics when a patient expects it.	☐	☐	☐	☐	☐	☐	☐

	Yes	No	
5.3a I have experienced antibiotic prescription for myself or my kin.	☐	☐	➔ *Proceed to 5.5a*
5.3b When antibiotics are prescribed, the doctor takes time to provide information on how they should be used, in an understandable manner.	☐	☐	
5.4 I usually know how antibiotics should be taken, even if I was given information about their use.	☐	☐	
5.5a I have experience, as a patient or as kin, of acquiring prescribed antibiotics from a pharmacy.	☐	☐	➔ *Proceed to 5.6*
5.5b Pharmacy staff take their time to inform me on how antibiotics should be used.	☐	☐	

	Totally agree					Don’t agree at all	Don’t know
5.6 I often know before I visit a doctor, whether I need antibiotics or not.	☐	☐	☐	☐	☐	☐	☐
5.7 I am confident in a doctor’s decision if s/he does not prescribe antibiotics.	☐	☐	☐	☐	☐	☐	☐
5.8 A doctor who does not prescribe antibiotics when the patient thinks that they are needed, is not as good a doctor.	☐	☐	☐	☐	☐	☐	☐
5.9 I am confident in a doctor's decision if s/he prescribes antibiotics.	☐	☐	☐	☐	☐	☐	☐
5.10 I usually know how infections should be treated.	☐	☐	☐	☐	☐	☐	☐
5.11 If I get an infection, I often wait and see, i.e. rest and take it easy, and see if the infection goes away on its own.	☐	☐	☐	☐	☐	☐	☐
5.12 Hand hygiene (hand washing or alcohol hand rub) reduces the risk of spreading common infections, such as influenza.	☐	☐	☐	☐	☐	☐	☐

	Yes	No
5.13 I usually get vaccinated against seasonal influenza.	☐	☐

	Totally agree					Don’t agree at all	Don’t know
5.14 I am confident in the work that Swedish healthcare in carrying out to minimise the development of resistance.	☐	☐	☐	☐	☐	☐	☐
5.15 I am confident in the work that the EU is carrying out to minimise the development of resistance.	☐	☐	☐	☐	☐	☐	☐
5.16 I am confident that pharmaceutical companies will be able to develop new medicines which will solve the problem of antibiotic resistance.	☐	☐	☐	☐	☐	☐	☐

5.17 Do you have any other comments/views about antibiotics, infections, resistance or how to prevent infection?

____

____

____

____

____

____

6. Background information

6.1 Sex

- ☐ Female
- ☐ Male

6.2 Age

________ years old

6.3 Education. *Please tick the highest level of the education that you have begun.*

- ☐ Primary and secondary school (or equivalent)
- ☐ Upper secondary school (or equivalent)
- ☐ University (or equivalent)

6.4 Country where you educated (for the most part).

____

6.5 Do you have a medical/healthcare-related education? *Regardless of level.*

- ☐ Yes. What education? ____________________
- ☐ No

6.6 Income. *Please tick only one option.*

- ☐ Up to 14,900 SEK/month
- ☐ 15,000-25,900 SEK/month
- ☐ 26,000-40,900 SEK/month
- ☐ More than 41,000 SEK/month

6.7 Post code

____
