# Supplementary material for: Knowledge and Attitudes towards Antibiotic Use and Resistance - A Latent Class Analysis of a Swedish Population-Based Sample
Source: PLoS One. 2016 Apr 20;11(4):e0152160. doi: 10.1371/journal.pone.0152160 (PMC4838333; doi:10.1371/journal.pone.0152160)
Supplement: S5 Appendix — (PDF) [file pone.0152160.s005.pdf]

Karolinska Institutet

Smi
SMITTSKYDDSINSTITUTET

Enkät om antibiotika

Volym 1 (2500 st): 0001–2500

Enkät om antibiotika

Du har blivit utvald att delta i en enkätstudie gällande uppfattningar och förväntningar i samband med användning av antibiotika.

Varför görs studien?
Antibiotika är en läkemedelsgrupp som används för att behandla infektioner. För att antibiotika ska kunna användas på ett bra sätt är det viktigt att veta vilka uppfattningar och förväntningar som finns bland allmänheten. Studien genomförs i samarbete mellan Karolinska Institutet och Smittskyddsinstitutet. Karolinska Institutet är forskningshuvudman.

Varför har just du blivit utvald?
Du är en av 2500 personer i landet mellan 18 och 74 år som har blivit slumpmässigt utvald för att medverka i studien. Adressuppgifterna är hämtade från SPAR-registret (Statens personadressregister) som är ett offentligt register omfattande alla som är folkbokförda i Sverige. Din medverkan är helt frivillig och du har rätt att avbryta din medverkan när som helst. Dina svar är mycket värdefulla för oss och Du kan inte ersättas av någon annan. Ditt deltagande bidrar till att resultatet blir tillförlitligt. Det är lika viktigt att Du deltar även om Du aldrig använt antibiotika.

Vad händer med svaren?
Svaren som lämnas kommer att bearbetas och sammanställas helt anonymt. Informationen skyddas enligt offentlighets- och sekretesslagen (2009:400) och personuppgiftslagen (1998:204). Alla som arbetar med studien har tystnadsplikt och insamlade uppgifter redovisas i tabeller där enskilda personers svar inte kan utläsas. Alla identitetsuppgifter kommer att avlägsnas.

Hur redovisas svaren?
Resultaten från studien kommer att användas för Smittskyddsinstitutet kunskapsspridning via exempelsvis faktabroschyrer gällande antibiotikaanvändning. Dessutom kommer de att presenteras i en artikel i en vetenskaplig tidskrift och eventuellt i en doktorsavhandling.

Har du några frågor?
Om Du har några frågor som rör studien eller vill ansöka om information (så kallat registerutdrag) och få rättelse av eventuellt felaktiga personuppgifter är Du välkommen att kontakta Professor Cecilia Stålsby Lundborg eller Sjuksköterska Martina Vallin. Om Du i framtiden skulle bli sjuk ska Du följa de råd Din läkare ger Dig.

If you don't speak Swedish
You have been selected to participate in a survey regarding antibiotics. If you don't speak Swedish and therefore can't answer the questions, you can take help of someone else to answer the questionnaire. You can also contact us and we will try to help you.

Personuppgiftansvarig: Karolinska Institutet

Cecilia Stålsby Lundborg
Professor
Inst. för folkhälsovetenskap
Karolinska Institutet
E-post: cecilia.stalsby.lundborg@ki.se
Tel. 08-52483366

Martina Vallin
Leg Sjuksköterska, forskningsassistent
Inst. för folkhälsovetenskap
Karolinska Institutet
E-post: martina.vallin@ki.se
Tel. 08-52483375

Aase Sten
Kommunikatör
Smittskyddsinstitutet
E-post: aase.sten@smi.se
Tel. 070-338 23 32,
08-4572332

13-02-13

Instruktioner

När ska jag fylla i enkäten?
Vi önskar att Du fyller i enkäten så snart som möjligt, helst inom ett par dagar. Lägg den ifyllda enkäten i det bifogade svarskuvertet. Portot är betalt.

Jag har råkat tappa bort svarskuvertet/enkäten, vad ska jag göra?
Du kan invänta en påminnelse då en ny enkät och ett svarskuvert skickas ut. Du kan även skicka ett email eller ringa en av kontaktpersonerna och ange Ditt löpnummer som står högst upp på enkätens fram- och baksida så skickar vi en ny enkät/svarskuvert till Dig.

Vad gör man om den person som fått en enkät hemskickad har flyttat, är bortrest eller är avliden?
Om personen som fått enkäten är bortrest men kommer tillbaka senast den 1 mars kan hon/han fylla i och skicka in enkäten då. Om personen inte är tillbaks innan dess, eller har flyttat eller är avliden skulle vi vara tacksamma om Du mailade eller ringde oss och uppgav löpnumret som står högst upp på enkätens fram- och baksida och eventuell ny adress.

Vad ska jag göra om jag inte vill fylla i enkäten?
Om Du inte vill svara på enkäten och vill slippa få påminnelser kan Du skicka ett email eller ringa en av kontaktpersonerna och ange Ditt löpnummer så tar vi bort Dig från undersökningen. Vi är tacksamma att Du meddelar oss om Du inte skulle vilja fylla i enkäten. Du behöver inte ange skäl till detta.

Löpnumret
Löpnumret är det 5- siffriga numret som står högst upp på enkätens fram- och baksida.

Kontaktpersoner
Cecilia Stålsby Lundborg, cecilia.stalsby.lundborg@ki.se, tel. 08-52483366
Martina Vallin, martina.vallin@ki.se, tel. 08-52483375

Våra möjligheter att svara i telefon är begränsade och det är därför bättre om Du i första hand kontaktar oss per email.

Hur ska jag fylla i enkäten:

Fyll i enkäten med en kulspetspenna. Markera svaren med ett kryss ☒. Om Du vill ändra ditt svar täcker du över den felaktiga rutan ■ och sätter ett kryss i rutan för det svar Du vill ange.

1. Antibiotikaanvändning

Antibiotika är ett läkemedel som ibland används för att behandla infektioner. Det finns flera olika typer av antibiotika, penicillin är den vanligaste sorten.

1.1 Vilka antibiotika känner du till?

____

____

1.2 Vilka av följande läkemedel är antibiotika? *Du kan kryssa i flera alternativ.*

- ☐ Paracetamol
- ☐ Seloken
- ☐ Kåvepenin
- ☐ Bricanyl
- ☐ Selexid
- ☐ Alvedon

1.3 Har du någon gång använt antibiotika? *Kryssa endast i ett alternativ.*

- ☐ Ja
- ☐ Nej ➔ *Gå vidare till 1.5*
- ☐ Vet ej ➔ *Gå vidare till 1.5*

1.4 Hur många gånger har du använt antibiotika de senaste 12 månaderna? *Kryssa endast i ett alternativ.*

- ☐ 0 gånger
- ☐ En gång
- ☐ 2-5 gånger
- ☐ Mer än 5 gånger

1.5 Hur många gånger har någon annan vuxen än du (person över 18 år) i hushållet fått antibiotika de senaste 12 månaderna? *Kryssa endast i ett alternativ.*

- ☐ 0 gånger
- ☐ 1 gång
- ☐ 2-5 gånger
- ☐ Mer än 5 gånger
- ☐ Finns ingen annan vuxen
- ☐ Vet ej

1.6 Hur många barn under 18 år finns det som bor regelbundet (deltid eller mer) i hushållet? *Kryssa endast i ett alternativ.*

- ☐ 0 ➔ *Gå vidare till 1.8*
- ☐ 1
- ☐ 2
- ☐ 3 eller flera

1.7 Hur många gånger har barnen i hushållet fått antibiotika sammanlagt de senaste 12 månaderna? *Kryssa endast i ett alternativ.*

- ☐ 0 gånger
- ☐ 1 gång
- ☐ 2-5 gånger
- ☐ Mer än 5 gånger
- ☐ Vet ej

1.8 Tar någon i hushållet antibiotika just nu? *Kryssa endast i ett alternativ.*

- ☐ Ja, för vad? ______________________ Om ja, ange ålder på denna person ________ år
- ☐ Nej
- ☐ Vet ej

2. Tillgång till antibiotika

Följande frågor besvaras med en skala. Markera med hjälp av skalan i vilken grad du instämmer i frågan/påståendet. Kryssa endast i ett alternativ.

	Håller fullständigt med					Tar helt avstånd ifrån	Vet ej
2.1 Antibiotika som blivit över kan man spara för att själv kunna ta senare eller för att ge till någon annan.	☐	☐	☐	☐	☐	☐	☐
2.2 Antibiotika som blivit över ska man ta med sig tillbaka till apoteket.	☐	☐	☐	☐	☐	☐	☐
2.3 Jag tycker att det är bra att man behöver ett recept för att kunna hämta ut antibiotika på apotek i Sverige.	☐	☐	☐	☐	☐	☐	☐
2.4 Jag tycker att det är bra att kunna köpa antibiotika via internet, utan att behöva träffa läkare.	☐	☐	☐	☐	☐	☐	☐
2.5 Jag tycker att det är bra att kunna få antibiotika av släktingar eller bekanta, utan att behöva bli undersökt av en läkare.	☐	☐	☐	☐	☐	☐	☐
2.6 Det är bra att man kan köpa antibiotika utan recept på apotek i vissa länder.	☐	☐	☐	☐	☐	☐	☐

3. Användningsområde och effekt

	Håller fullständigt med					Tar helt avstånd ifrån	Vet ej
3.1 Antibiotika gör att man blir fortare frisk vid en förkylning.	☐	☐	☐	☐	☐	☐	☐
3.2 Om snuvan vid en förkylning blir färgad behöver man alltid antibiotika för att bli av med snuvan.	☐	☐	☐	☐	☐	☐	☐
3.3 Lindringa infektioner klarar kroppen oftast av själv utan antibiotika.	☐	☐	☐	☐	☐	☐	☐
3.4 Hosta som varar längre än en vecka behöver alltid behandlas med antibiotika för att försvinna.	☐	☐	☐	☐	☐	☐	☐
3.5 Det är lämpligt att använda antibiotika vid halsont och förkylning eftersom man annars kan få följdsjukdomar.	☐	☐	☐	☐	☐	☐	☐
3.6 Det är lämpligt att använda antibiotika vid halsfluss eftersom man annars kan få följdsjukdomar.	☐	☐	☐	☐	☐	☐	☐

	Håller fullständigt med					Tar helt avstånd ifrån	Vet ej
3.7 En öroninflammation hos ett barn som är 3-6 år gammalt behöver alltid behandlas med antibiotika.	☐	☐	☐	☐	☐	☐	☐
3.8 En blåskatarr/nedre urinvägsinfektion hos en kvinna kan läka ut även utan antibiotika.	☐	☐	☐	☐	☐	☐	☐
3.9 Genom att använda antibiotika kan man ofta undvika att vara hemma från jobbet.	☐	☐	☐	☐	☐	☐	☐
3.10 Antibiotika ska döda alla bakterier i kroppen.	☐	☐	☐	☐	☐	☐	☐

4. Bieffekter och resistens

	Ja	Nej	Vet ej
4.1 Antibiotika ger ofta biverkningar som t.ex. diarré.	☐	☐	☐
4.2 Antibiotika ger negativa effekter på kroppens egen bakterieflora.	☐	☐	☐
4.3 Om man känner sig frisk efter delar av en antibiotikakur kan man genast avsluta kuren.	☐	☐	☐
4.4 Bakterier kan bli resistenta mot antibiotika.	☐	☐	☐
4.5 Ju mer antibiotika vi använder i samhället desto större risk är det att resistens utvecklas och sprids.	☐	☐	☐
4.6 Människor kan bli resistenta mot antibiotika.	☐	☐	☐
4.7 Användning av antibiotika bland djur kan minska möjligheten till effektiv antibiotikabehandling bland människor.	☐	☐	☐
4.8 Resistens kan spridas från djur till människa.	☐	☐	☐
4.9 Resistens kan spridas från människa till människa.	☐	☐	☐
4.10 Utlandsresenärer riskerar att ta med sig resistens hem till Sverige.	☐	☐	☐

	Håller fullständigt med					Tar helt avstånd ifrån	Vet ej
4.11 Antibiotikaresistens är ett stort problem i Sverige idag.	☐	☐	☐	☐	☐	☐	☐
4.12 Antibiotikaresistens är ett stort problem i övriga världen idag.	☐	☐	☐	☐	☐	☐	☐

5. Läkarvanor och patient/läkarrelation samt prevention

	Håller fullständigt med					Tar helt avstånd ifrån	Vet ej
5.1 Läkare undersöker alltid ordentligt om patienten behöver antibiotika eller inte.	☐	☐	☐	☐	☐	☐	☐
5.2 Läkare skriver ut antibiotika för att patienten förväntar sig det.	☐	☐	☐	☐	☐	☐	☐

	Ja	Nej	
5.3a Jag har erfarenhet av att jag eller någon anhörig fått antibiotika utskrivet.	☐	☐	➔ *Gå vidare till 5.5a*
5.3b När antibiotika förskrivs tar sig läkare tid vid besöket att informera så man förstår hur antibiotikan ska användas.	☐	☐	
5.4 Jag vet oftast hur antibiotika ska användas även om jag inte informerats om användningen.	☐	☐	
5.5a Jag har erfarenhet av att som patient eller anhörig hämta utskrivet antibiotika på apotek.	☐	☐	➔ *Gå vidare till 5.6*
5.5b Apotekspersonal tar sig tid att informera hur antibiotikan ska användas.	☐	☐	

	Håller fullständigt med					Tar helt avstånd ifrån	Vet ej
5.6 Jag vet ofta själv om jag behöver antibiotika eller inte innan jag träffar läkaren.	☐	☐	☐	☐	☐	☐	☐
5.7 Jag känner förtroende för läkarens beslut om läkaren avstår från att skriva ut antibiotika.	☐	☐	☐	☐	☐	☐	☐
5.8 En läkare som inte skriver ut antibiotika när patienten tycker att det behövs, är en mindre bra läkare.	☐	☐	☐	☐	☐	☐	☐

	Håller fullständigt med					Tar helt avstånd ifrån	Vet ej
5.9 Jag känner förtroende för läkarens beslut om läkaren skriver ut antibiotika.	☐	☐	☐	☐	☐	☐	☐
5.10 Jag vet oftast själv hur infektioner ska behandlas.	☐	☐	☐	☐	☐	☐	☐
5.11 Jag avvaktar oftast om jag får en infektion, dvs vilar och tar det lugnt och ser om infektionen ger med sig av sig själv.	☐	☐	☐	☐	☐	☐	☐
5.12 Handhygien (handtvätt eller handsprit) minskar risken för spridning av vanliga infektioner, t.ex. influensa.	☐	☐	☐	☐	☐	☐	☐

	Ja	Nej
5.13 Jag brukar vaccinera mig mot säsongsinfluensa.	☐	☐

	Håller fullständigt med					Tar helt avstånd ifrån	Vet ej
5.14 Jag känner förtroende för den svenska sjukvårdens arbete för att minimera resistensutvecklingen.	☐	☐	☐	☐	☐	☐	☐
5.15 Jag känner förtroende för EUs arbete för att minimera resistensutvecklingen.	☐	☐	☐	☐	☐	☐	☐
5.16 Jag känner förtroende för att läkemedelsbolag kommer att kunna ta fram nya preparat som kommer att lösa problemen med antibiotikaresistens.	☐	☐	☐	☐	☐	☐	☐

5.17 Har du övriga synpunkter kring antibiotika, infektioner, resistens eller hur man kan förebygga infektioner?

____

____

____

____

____

____

6. Bakgrundsinformation

6.1 Kön

☐ Kvinna
☐ Man

6.2 Ålder

__________år

6.3 Utbildningsnivå. *Kryssa i högsta påbörjade nivå.*

☐ Grundskola eller motsvarande ☐ Gymnasium eller motsvarande
☐ Universitet/högskoleutbildning eller motsvarande

6.4 Land där huvuddelen av utbildningen genomfördes?

____

6.5 Har du någon vårdrelaterad/medicinsk utbildning? *Oavsett nivå.*

☐ Ja. Vilken utbildning? ______________________
☐ Nej

6.6 Inkomst. *Kryssa endast i ett alternativ.*

☐ Upp till 14,900 kr/ mån
☐ 15,000-25,900 kr/ mån
☐ 26,000 – 40,900 kr/ mån
☐ Mer än 41,000 kr/ mån

6.7 Postnummer

____

Volym 1 (2500 st): 0001–2500
